# Incidence and risk factor for short term postoperative cough after thyroidectomy

**DOI:** 10.1186/s12885-020-07392-1

**Published:** 2020-09-16

**Authors:** Junfu Wu, Liyuan Dai, Weihua Lou

**Affiliations:** 1 Department of Otolaryngology-Head and Neck Surgery, The First Affiliated Hospital of Zhengzhou University, Zhengzhou, 450085 P.R. China; 2 Department of Head and Neck Surgery, Henan Cancer Hospital, Zhengzhou, 450008 P.R. China

**Keywords:** Acute cough, Leicester cough questionnaire, Thyroidectomy, Thyroid cancer, Postoperative cough

## Abstract

**Background:**

The prevalence of potential risk factors for postoperative cough after thyroidectomy remain unknown. The current study aimed to research postoperative cough in patients undergoing thyroid surgery prospectively.

**Methods:**

Adult patients who underwent primary thyroid surgery were selected prospectively. Data regarding age, sex, BMI, pathology and surgical procedure were collected and analyzed. The Leicester Cugh Questionnaire (LCQ) was required to be completed by all patients before operation, 2 weeks and 4 weeks after operation.

**Results:**

There were 1264 patients enrolled in total. Eleven patients with vocal cord paralysis were excluded. In patients with benign disease, postoperative cough occurred in 61 patients, with an prevalence rate of 17. 0% compared to an prevalence rate of 33.1% in patients with malignant disease; the difference was significant. For benign patients, the factors of smoking and operation time were independently related to the occurrence of postoperative cough. For malignant patients, the factors of smoking, operation time, operation extent, and the number of positive nodes at level 6 were independently related to the occurrence of postoperative cough. There was no significant difference regarding the LCQ score in patients with benign or malignant disease at the preoperative and the postoperative 4-week time periods. Patients with malignant disease had a significantly lower LCQ score than patients with benign disease at the postoperative 2-week time point (*p* = 0.004).

**Conclusions:**

Patients undergoing thyroid cancer surgery had a higher incidence of postoperative cough and were also associated with a decreased cough-related quality of life. The factors of smoking and operation time were the most important predictors for postoperative cough after thyroidectomy.

## Background

There has been a substantial increase in the proportion of thyroid cancer cases globally, on the one hand, because the prevalence has really increased, on the other hand, because of the prevalence of thyroid color Doppler ultrasound examination [1]. Usually the disease is asymptomatic, surgery is the first choice of treatment, and thyroidectomy is considered as an effective and safe option for most patients who have low chance of suffering permanent vocal distortion, swallowing difficulties, and hypocalcemia when performed by experienced surgeons [2–4]; however, some postoperative symptoms such as nausea and vomiting, local sensory disturbance and transient throat pain are still inevitable after the surgery. In our cancer center, some patients who have done thyroidectomy may also suffer serious cough; postoperative cough can even induce postoperative hematoma [2]. Patients complain that this phenomenon is quite worrisome, and doctor-patient conflicts may even occur [5], especially in patients without a previous history of cough. This suggests the importance of preoperative communication for postoperative cough. Factors including smoking history, surgical extent, and operation time might be associated with postoperative cough. However, there are few studies available in terms of its prevalence and potential predictors.

Since its first introduction by Birring et al. [6], the Leicester Cough Questionnaire (LCQ) has been regarded as a reliable tool for evaluating the cough in adults by a number of researchers [7–9] . Therefore, our goal was to prospectively analyze the postoperative cough in patients who undergoing thyroidectomy.

## Methods

Ethics approval and consent to participate: Henan Cancer Hospital Research Ethics committee (approval number: HNZZ20170102) approved this study, written informed consent was obtained from all patients at initial treatment.

From January 2018 to December 2018, adult (≥18 years) patients undergoing primary thyroidectomy were prospectively tracked. The exclusion criteria were as follows: the patient had chronic cough associated with smoking or gastroesophageal reflux or with other causes; resection of the trachea or larynx was performed; the recurrent laryngeal nerve was invaded by the tumor or metastatic nodes resulting in recurrent laryngeal nerve paralysis; and there was pulmonary infection. The symptom of cough had to begin on the first day after the operation, and was defined and assessed by our research group based on previous studies [10]. Patients who had a history of smoking/drinking at the time of diagnosis or had quit smoking/drinking for less than 1 year were defined as smokers/drinkers [11]. The operation time was defined as “the time from the beginning of endotracheal intubation to the point of extubation” [12]. Data regarding age, sex, BMI, operation time, postoperative pathology, operation type, and drinking and smoking status were collected and analyzed. Based on the Chinese Nutrition Society, overweight refers to a BMI from 24 to 28, and obesity refers to a BMI above 28.

All patients received an open surgery under general anesthesia using both intravenous anesthesia and inhalation anesthesia, the frequently used narcotic drugs included cisatracurium, propoxate and fentanyl. After the operation, patients needed to stay for a short time in the recovery room and then they would be transferred to the ward. The extent of operation of the primary tumor consisted of two types: unilateral thyroid operation referred to surgery involving only one thyroid lobe, and bilateral thyroid operation referred to surgery involving both thyroid lobes. In our hospital, central neck dissection was routinely performed for thyroid papillary and medullary carcinoma. Lateral neck dissection was performed if there were positive nodes at level 3 or 4 according to frozen sections. All patients had atomised inhalation after surgical treatment.

All patients enrolled needed to complete the Mandarin Chinese Version of LCQ [13] preoperatively in the ward, and patients with postoperative cough were required to complete the LCQ at 2 weeks and 4 weeks postoperatively via the out-patient department, email, or WeChat. The LCQ was usually used for measuring chronic cough, but recent evidence showed there was also high validity and responsiveness in assessing acute cough or postoperative cough [7, 14, 15]. The LCQ is easy to complete taking less than 5 min by themselves. There are 19 items in total, each item represents an adverse event caused by cough. The responses were scored by a 7-point Likert scale. The 19 items were divided into three areas that considered the psychological effects (for instance the impact of cough on embarrassment/anxiety), physical effects (for instance the impact of cough on chest and stomach pain), and social effects (for instance the effect of cough on work/daily life and entertainment life). A total score and three domain scores were calculated, the score in each domain is between 1 and 7, and the total score is between 3 and 21; the higher the score, the better the health [16].

The data of continuous variables were represented as mean ± standard deviation (SD), and the classified variables were represented as frequency and percentage. A univariate analysis (the Chi-square test, t-test) was used to evaluate the possible risk factors for postoperative cough, and then a multivariate analysis (logistic regression test) was used to determine the independent risk factors. The Wilcoxon signed-rank test was used to compare the LCQ scores among different time periods. All statistical analyses were carried out by SPSS 20.0, and *p* < 0.05 was considered significant.

### Results

There were 1264 patients (922 females and 342 males) participated in the study, and the average age was 49.4 (range: 18–78) years, including 39 smokers and 35 drinkers. A total of 577 patients were considered to be overweight, and 171 patients were obese. The postoperative pathology was benign in 361 patients and malignant in 903 patients. The mean operation time was 1.6 (range: 0.7–4.8) hours. There were 19 cases of postoperative hemorrhage, 186 cases of transient hypocalcemia and 11 cases of vocal cord paralysis. Patients with vocal cord paralysis were excluded.

A total of 357 patients had postoperative cough, and the overall prevalence was 28.5%. In patients with cough, 6 developed postoperative bleeding, and in patients without cough, 13 developed postoperative bleeding; the statistical difference was not significant (*p* = 0.764). In patients with benign disease, postoperative cough occurred with an prevalence rate of 17.0%, in these patients, 2 (3.2%) patients had postoperative bleeding, 4 (6.6%) patients had transient hypocalcemia, in patients without cough, 2 (0.7%) patients had postoperative bleeding, 20 (6.7%) patients had transient hypocalcemia, the mean operation time was 1.3 (range: 0.7–2.4) hours.

In patients with malignant disease, postoperative cough occurred with an prevalence rate of 33.1%, in these patients, 4 (1.4%) patients had postoperative bleeding, 42 (14.2%) patients had transient hypocalcemia, in patients without cough, 11 (1.8%) patients had postoperative bleeding, 120 (20.1%) patients had transient hypocalcemia, and the mean operation time was 1.6 (range: 0.8–4.8) hours.

The differences regarding cough occurrence and operation time between patients with benign and malignant tumors were both significant (both *p* < 0.001). There were no statistical differences in age, sex, or BMI between the two groups (all *p* > 0.05).

To find out the risk factors of postoperative cough in patients with benign disease, as described in Table 1, in the univariate analysis, the factors of smoking, operation time, and operation extent were associated with the occurrence of postoperative cough (all *p* < 0.05). In further multivariate logistic regression analysis (Table 2), the factors of smoking and operation time were related to the occurrence of postoperative cough (all *p* < 0.05).

**Table 1:** Univariate analysis of risk factors for postoperative cough in patients with benign thyroid disease

Variables	Univariate		
	Cough (*n* = 61)	No cough (*n* = 298)	
Age (year)	50.23 ± 7.34	48.82 ± 7.05	0.203
Sex			
Female	47(13.1%)	200(55.7%)	
Male	14(3.9%)	98(27.3%)	0.127
Smoker			
No	53(14.8%)	288(80.2%)	
Yes	8(2.2%)	10(2.8%)	0.001
Drinker			
No	56(15.6%)	288(80.2%)	
Yes	5(1.4%)	10(2.8%)	0.085
Operation time (hour)	1.42 ± 0.83	1.24 ± 0.61	0.006
BMI			
Normal	27(7.5%)	103(28.7%)	
24 ~ 28	23(6.4%)	129(35.9%)	
> 28	11(3.1%)	66(18.4%)	0.352
Operation extent Unilateral	24(6.7%)	166(46.2%)	
Bilateral	37(10.3%)	132(36.8%)	0.020

**Table 2:** Multivariate analysis of risk factors for postoperative cough in patients with benign thyroid disease

Variables	Multivariate analysis	
	p	OR [95% CI]
Smoker	0.011	3.323 [1.531–7.769]
Operation time	0.004	1.851 [1.186–4.373]
Operation extent	0.464	2.768 [0.656–5.108]

To find out the risk factors of postoperative cough in patients with malignant disease, as described in Table 3, in the univariate analysis, the factors of smoking, operation time, operation extent, the number of positive nodes at level 6, and lateral neck dissection were associated with the occurrence of postoperative cough (all *p* < 0.05). In further multivariate logistic regression analysis (Table 4), the factors of smoking, operation time, operation extent, and the number of positive nodes at level 6 were related to the occurrence of postoperative cough (all *p* < 0.05).

**Table 3:** Univariate analysis of risk factors for postoperative cough in patients with malignant thyroid disease

Variables	Univariate		
	Cough (*n* = 296)	No cough (*n* = 598)	
Age (year)	50.09 ± 8.12	48.16 ± 7.96	0.261
Sex			
Female	213(23.8%)	455(50.9%)	
Male	83(9.3%)	143(16.0%)	0.181
Smoker			
Yes	11(1.2%)	9(1.0%)	
No	285(31.9%)	589(65.9%)	0.017
Drinker			
Yes	10(1.1%)	9(1.0%)	
No	286(32.0%)	589(65.9%)	0.068
Operation time (hour)	1.82 ± 0.84	1.65 ± 0.78	0.001
BMI			
Normal	116(13.0%)	266(29.8%)	
24 ~ 28	140(15.6%)	280(31.3%)	
> 28	40(4.5%)	52(5.8%)	0.051
Operation extent			
Unilateral	83(9.3%)	306(34.2%)	
Bilateral	213(23.8%)	292(32.7%)	< 0.001
Number of positive nodes in level 6			
≥ 3	117(13.1%)	189(21.1%)	
< 3	179(20.0%)	409(45.8%)	0.019
Lateral neck dissection			
Yes	98(11.0%)	250(28.0%)	
No	198(22.1%)	348(38.9%)	0.012
Cancer type			
PTC^a^	286(32.0%)	571(63.9%)	
others	10(1.1%)	27(3.0%)	0.422

^a^*PTC* papillary carcinoma

**Table 4:** Multivariate analysis of risk factors for postoperative cough in patients with malignant thyroid disease

Variables	Multivariate analysis	
	p	OR [95% CI]
Smoker	0.004	4.102 [1.668–8.476]
Operation time	0.002	3.401 [1.346–7.051]
Operation extent	0.016	2.976 [1.245–4.796]
Number of positive nodes in level 6	< 0.001	5.701 [2.021–9.501]
Lateral neck dissection	0.087	2.428 [0.879–7.492]

**Fig. 1:**
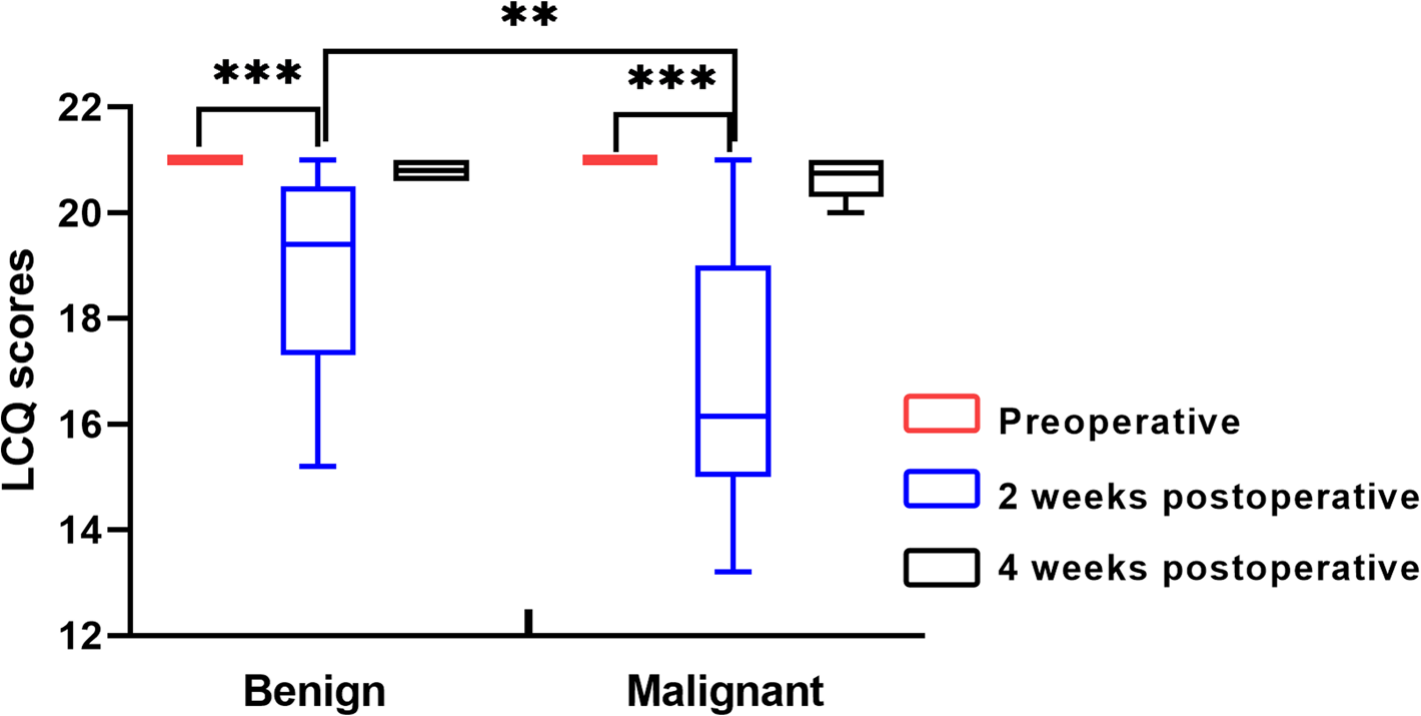
Changes of LCQ scores in different groups at different time periods: ** *p* < 0.01, *** *p* <0.001

In coughing patients with benign disease, the mean preoperative LCQ score was 21, and the mean LCQ score was 18.8 (SD: 3.6) at the second week after the operation; the difference was significant (Fig. 1, *p* < 0.001). The mean LCQ score was 20.8 (SD: 0.2) at the fourth week after the operation, and when compared to the preoperative level, the difference was not significant (*p* = 0.706).

In coughing patients with malignant disease, the mean preoperative LCQ score was 21, and the mean LCQ score was 16.7 (SD: 5.9) the second week after the operation; the difference was significant (Fig. 1, *p* < 0.001). The mean LCQ score was 20.7 (SD: 0.4) 4 weeks postoperatively, and when compared to the preoperative level, the difference was not significant (*p* = 0.731).

When comparing the scores among different time periods in patients with benign or malignant disease, there was no statistically significant difference between the two groups at the preoperative and postoperative 4-week time periods (both *p* > 0.05), but patients with malignant disease had significantly lower LCQ scores than patients with benign disease at the postoperative 2-week time period (Fig. 1, *p* = 0.004).

## Discussion

Recurrent laryngeal nerve injury and parathyroid injury are common complications after thyroidectomy and common causes of doctor-patient conflict [17, 18]. However, with the increasing demand for high-quality medical treatment, severe postoperative cough has become an aspect of concern in recent years. Our findings have shown that postoperative cough was relatively common after thyroid surgery, with an overall prevalence rate of 28.5%, and it was more common in patients with malignant disease than in patients with benign disease. In a study by Jung et al. [19], the author’s goal was to assess the effect of a humidifier with heated wire circuits on the prevalence and severity of cough after thyroid surgery, and they found that in patients undergoing active humidification of inspired gases, the prevalence of postoperative cough was significantly decreased compared to patients without a heated humidifier. In the current study, all patients had routine postoperative aerosol inhalation of budesonide aerosol and ambroxol, twice a day for 5 days. The overall prevalence of postoperative cough was in agreement with the findings of the above study.

The prevalence of postoperative cough in patients with malignant diseases was higher than that in patients with benign diseases. This finding was interesting. Most previous authors have analyzed cough in patients with a flexible reinforced laryngeal mask airway or a plain endotracheal tube [20–22]; although they described that compared with conventional endotracheal intubation for the placement of flexible reinforced laryngeal mask airway during operation can reduce the incidence and severity of laryngo-pharyngeal symptoms, no authors have evaluated whether the type of pathology affects postoperative cough. The variation between the two groups might be explained by the following: surgical trauma, anesthesia, tracheal intubation, and recurrent laryngeal nerve dissection are all potential causes for cough following surgical procedures [23], and patients with malignant disease had longer operation times and more instances of routine central neck dissection in the current study.

Risk factors for postoperative cough following surgical procedures have been occasionally analyzed. Lin et al. [12] demonstrated that a long duration of anesthesia time, female sex, subcarinal node resection and lower paratracheal node resection were independent risk factors for postoperative cough in non-small cell lung cancer patients. The correlation between lung surgery and postoperative cough was reported by Chen et al. [24]. They found that the more aggressive patients were, the higher the probability of postoperative cough. This study was the first to analyze the predictors of postoperative cough after thyroidectomy. Similar to previous reports, we also noted that operation time was an independent predictor for postoperative cough. Moreover, smoking has been proven to be related to chronic cough by Colak et al. [25], and there was also a positive linkage with acute cough based on our outcome.

Another interesting finding was that postoperative cough was more common in patients with more than 3 positive central nodes. In our view, the most likely cause of this finding is associated with the branches of the recurrent laryngeal nerve. Small branches of the nerve, such as the tracheal branch, are often encountered during thyroidectomy, and they may inadvertently be resected during central neck lymph node dissection. However, the actual frequency of branch excision has not been clearly documented. More research is needed to clarify this issue. Another potential explanation is the tracheal thermal damage associated with the usage of high-frequency electric surgical knives and ultrasonic scalpels during operation.

It is important to evaluate the impact of postoperative cough on quality of life. The LCQ is a reliable method that has been used as an outcome measure in many clinical trials [15, 23, 26]. Lin et al. [12] described that in patients receiving video-assisted thoracoscopic surgery for lung cancer, the mean postoperative total score was 16.35, which was significantly lower than the mean follow-up score after 1 month, but the authors did not provide the data of preoperative levels. In our previous study, we found that thyroidectomy was significantly associated with a decreased LCQ score compared to baseline scores, but the study did not report when the LCQ scores returned to preoperative levels. In the current study, we were the first to note that for patients undergoing thyroidectomy regardless of the presence of benign or malignant disease, the mean postoperative LCQ score returns to baseline level in 4 weeks. The time interval found in this study was significantly shorter than in patients undergoing lung surgery, which can be attributed to the different types of operation.

Moreover, we found that the 2-week mean LCQ score was quite lower in patients with malignant disease than in those with benign disease. One possible reason for this difference is that patients with malignant disease were associated with a longer operation time and more surgical trauma, including routine dissection of the recurrent laryngeal nerve.

We must admit that the study can be limited. Firstly, cough assessment usually consists of objective and subjective measures. Although the LCQ is a reliable method for subjective assessment, more objective analyses are needed to clarify postoperative cough after thyroidectomy. Secondly, related intubation laryngitis, or laryngeal trauma can also cause coughing after surgery. However, for patients with cough, endoscopy is not used for routine laryngeal examination. Any misclassification would deviate our analysis. Last but not the least, the BMI and lifestyle characteristics were significantly different in this group to a western group, it remained unclear whether this finding could be confirmed in western studies.

## Conclusions

In summary, compared to patients with benign disease, patients undergoing thyroid cancer surgery had a higher prevalence of postoperative cough and a lower 2-week postoperative LCQ score. However, the 4-week postoperative LCQ score returned to the preoperative level in patients undergoing thyroidectomy regardless of the presence of benign or malignant disease. The factors of smoking and operation time were the most important predictors for postoperative cough after thyroidectomy.

## Data Availability

The datasets used and/or analysed during this study could be achieved from the corresponding author.

## Abbreviations

LCQ: Leicester Cough Questionnaire
PTC: Papillary Carcinoma
